# Magnetic Resonance of Rectal Cancer Response to Therapy: An Image Quality Comparison between 3.0 and 1.5 Tesla

**DOI:** 10.1155/2020/9842732

**Published:** 2020-10-10

**Authors:** Damiano Caruso, Marta Zerunian, Domenico De Santis, Tommaso Biondi, Pasquale Paolantonio, Marco Rengo, Davide Bellini, Riccardo Ferrari, Maria Ciolina, Elena Lucertini, Michela Polici, Elsa Iannicelli, Vincenzo Tombolini, Andrea Laghi

**Affiliations:** ^1^Department of Medical-Surgical and Translational Medicine-Radiology Unit, “Sapienza” University of Rome, Sant'Andrea Hospital, Rome, Italy; ^2^Department of Radiological, Oncological and Pathological Sciences, “Sapienza” University of Rome, ICOT Hospital, Latina, Italy; ^3^Department of Radiotherapy, “Sapienza” University of Rome, Policlinico Umberto I, Rome, Italy

## Abstract

**Purpose:**

To evaluate signal intensity (SI) differences between 3.0 T and 1.5 T on T2-weighted (T2w), diffusion-weighted imaging (DWI), and apparent diffusion coefficient (ADC) in rectal cancer pre-, during, and postneoadjuvant chemoradiotherapy (CRT).

**Materials and Methods:**

22 patients with locally advanced rectal cancer were prospectively enrolled. All patients underwent T2w, DWI, and ADC pre-, during, and post-CRT on both 3.0 T MRI and 1.5 T MRI. A radiologist drew regions of interest (ROIs) of the tumor and obturator internus muscle on the selected slice to evaluate SI and relative SI (rSI). Additionally, a subanalysis evaluating the SI before and after-CRT (*∆*SI pre-post) in complete responder patients (CR) and nonresponder patients (NR) on T2w, DWI, and ADC was performed.

**Results:**

Significant differences were observed for T2w and DWI on 3.0 T MRI compared to 1.5 T MRI pre-, during, and post-CRT (all *P* < 0.001), whereas no significant differences were reported for ADC among all controls (all *P* > 0.05). rSI showed no significant differences in all the examinations for all sequences (all *P* > 0.05). *∆*SI showed significant differences between 3.0 T and 1.5 T MRI for DWI-*∆*SI in CR and NR (188.39 ± 166.90 vs. 30.45 ± 21.73 and 169.70 ± 121.87 vs. 22.00 ± 31.29, respectively, all *P* 0.02) and ADC-*∆*SI for CR (−0.58 ± 0.27 vs. −0.21 ± 0.24*P* value 0.02), while no significant differences were observed for ADC-*∆*SI in NR and both CR and NR for T2w-*∆*SI.

**Conclusion:**

T2w-SI and DWI-SI showed significant differences for 3.0 T compared to 1.5 T in all three controls, while ADCSI showed no significant differences in all three controls on both field strengths. rSI was comparable for 3.0 T and 1.5 T MRI in rectal cancer patients; therefore, rectal cancer patients can be assessed both at 3.0 T MRI and 1.5 T MRI. However, a significant DWI-*∆*SI and ADC-∆SI on 3.0 T in CR might be interpreted as a better visual assessment in discriminating response to therapy compared to 1.5 T. Further investigations should be performed to confirm future possible clinical application.

## 1. Introduction

In the last decade, magnetic resonance imaging (MRI) has assumed a crucial role in the local staging of primary and recurrent rectal cancer. Due to its high spatial and contrast resolution, MRI allows not only for tumor and nodal staging but also provides essential information, such as extramural vascular invasion and grade of tumor regression, which help in assessing disease-free survival and overall survival rates [1, 2].

Several studies have extensively investigated the role of 1.5-Tesla (T) MRI in rectal cancer as a robust and accurate imaging modality for preoperative local staging [3–8]. MRI is particularly accurate in assessing the distance between the tumor and the mesorectal fascia with sensitivity and specificity up to 94% and 76%, respectively [9, 10]. Theoretically, 3.0 T MRI achieves a twofold increase of signal-to-noise ratio (SNR) compared to 1.5 T systems due to the dependence of MRI signal to the magnetic field strength, resulting in improved spatial and temporal resolution [11–14].

Few studies have assessed patients with rectal cancer by means of 3.0 T MRI and 1.5 T MRI [11, 12, 15, 16]. In particular, Maas et al. [15] have shown that 3.0 T and 1.5 T MRI scanners have similar staging performances independently from the reader expertise; nevertheless, more studies ought to be performed, in particular evaluating quantitative imaging parameters to deepen the comparison between the two field strengths.

Thus, the aim of this work is to compare image quality of 1.5 T and 3.0 T in terms of signal intensity (SI) values in T2-weighted (T2w), DWI sequences, and apparent diffusion coefficient maps (ADC) in some patients with rectal cancer pre-, during, and postneoadjuvant chemoradiotherapy (CRT).

## 2. Materials and Methods

### 2.1. Study Population

After the approval of institutional ethics committee, all patients selected have accepted and signed the written informed consent.

Within the Italian Association for Cancer Research (AIRC) prospective trial study “MR Imaging Biomarkers in Response Evaluation to Neoadjuvant Chemoradiotherapy in Rectal Cancer” I.G. 2013, 90 consecutive nonrandomized patients were enrolled between February 2014 and October 2016. For the specific purpose of the study, we included 22 patients who accepted to undergo a second MRI.

Inclusion criterion was the presence of histologically confirmed stage II (cT3-4, N0, M0) or stage III (cT1-4, N+, M0) colorectal adenocarcinoma. Exclusion criteria were as follows: (a) implanted electrical devices or metallic foreign bodies incompatible with MRI, (b) incomplete MRI protocol or histopathological data, c) contraindication to the use of neoadjuvant CRT or surgical treatment or suspension of neoadjuvant treatment before undergoing surgery, (d) coexistence of other known tumors and/or previous pelvic radiotherapy, (e) proven medical history of hypersensitivity to the drug used in the study or to their excipients, and (f) simultaneous enrollment in other experimental clinical trials and consequent administration of experimental drugs within 30 days of joining this study.

### 2.2. Study Protocol

Patients underwent both 3.0 T and 1.5 T MRI examinations at three different moments of the trial, already described in another study part of the major project protocol [8]: (a) at the beginning for the locoregional staging, before treatment initiation; (b) during neoadjuvant CRT; and (c) at the end of CRT. To reduce time-related variability of the tumor burden, a time span of 24 hours was set to separate the two MRI examinations at each moment.

### 2.3. MR Examinations

All the MRI examinations were performed without bowel preparation, rectal distention, or antiperistaltic agents. Acquisitions were obtained in feet first supine position during free breathing. Detailed MRI protocol is reported in Table 1. 3.0 T MRI examinations were performed with a Discovery MR750 scanner (General Electrics, Milwaukee, WS, US), using 16-channel body coil. A routine imaging protocol for local staging of rectal cancer was performed including high-resolution T2-weighted fast recovery fast-spin echo sequence (2D FRFSE) (TR, 2086-4172 ms; TE, 11.4-122.3 ms; acceleration factor (Nex), 2; slice thickness, 4 mm; and matrix, 512 × 512 pixels) acquired on sagittal, transversal, and coronal axes. Additional oblique axial and coronal sequences were oriented, respectively, orthogonal and parallel to the long axis of the tumor. DWI sequences were also performed using a single-shot echoplanar imaging sequence with spectral adiabatic inversion recovery fat saturation technique (TR, 4400 ms; TE, 81.4 ms; Nex, 2; slice thickness, 4 mm; matrix, 256 × 256 pixels; and *b* values 0-200-600-800-1000 s/mm^2^), along the three orthogonal directions of the motion-probing gradients.

All patients underwent further MRI scan at a 1.5 T system Magnetom Avanto (Siemens Medical Solutions, Erlangen, Germany), using a 6-channel body coil. Routinely imaging protocol used for local staging of rectal cancer was applied including: oblique T2-weighted high-resolution images (axial and coronal)-oriented perpendicular and parallel to the tumor axis, respectively (TR, 3380 ms; TE, 90 ms; Nex, 2; slice thickness, 5 mm; and matrix, 366 × 448 pixels) and axial single-shot epi-DWI sequences (TR, 6000 ms; TE, 75 ms; Nex, 2; slice thickness, 5 mm; matrix, 168 × 224 pixels; and *b* values 0-50-400-800-1000 s/mm^2^). ADC maps in a gray scale were automatically extrapolated by the operating system. For the purpose of the study, only the oblique axial T2-weighted, DWI sequences, and ADC maps were analyzed.

### 2.4. Image Analysis

Images were analyzed by means of a workstation (Advantage Workstation 4.4, GE Healthcare, Milwaukee, WI) with a dedicated software (Volume Share 4), in a reporting room equipped with artificial environmental lighting (40 lx). An expert radiologists (AL) with 19 years of experience in oncologic gastrointestinal MRI imaging, with full access to clinical and histological patients' data, selected a single slice for sequence providing the best tumor depiction at oblique axial T2w, DWI, and ADC maps. The reader paid attention to select the same rectal level for each slice in order to maintain the same evaluation standard for the further analysis.

Once the slices were selected and anonymized, two expert radiologists in consensus (MC and DDS with 11 and 8 years of experience in rectal MRI, respectively) assessed the objective image quality blinded to the field strength, timeline of MRI examinations, and pathologic results. Images were grouped in stacks of 12 each and presented in random order for a total of 33 reading sections. A three-day time interval was set between each reading section.

**Figure 1:**
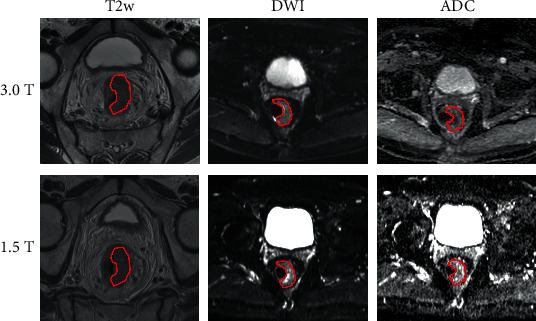
The picture shows manual segmentation of tumor region of interest in three different sequences (T2w, DWI, and ADC maps) on both field strengths, at the same slice.

A circular region of interest (ROI, mean size: 65 mm^2^; range, 50–75 mm^2^) was placed in consensus by the two readers in the lesions on T2w, DWI at 1000 *b* values, and ADC maps (Figure 1), carefully avoiding the rectal lumen, healthy rectal wall (in case of eccentric cancer), and mesorectal fat. For images with no obvious high SI representing the tumor, the three ROIs where placed within the apparent tumor where the residual tissue was visible even if hypointense and fibrotic. Additional circular ROIs (mean size: 65 mm^2^; range, 50–75 mm^2^) were placed in the right internus obturator muscle. All measurements were performed three times and eventually averaged. SI of each ROI was recorded, and relative SI (rSI) was normalized as follows:

(1) $$\begin{array}{c} \text{rSI}=\frac{{\text{SI}}_{\text{tumor}}}{{\text{SI}}_{\text{muscle}}}. \end{array}$$

Furthermore, according to pathology, a subanalysis on complete responders (CR) and nonresponders (NR) was performed, calculating a *∆* between the tumor SI before and after CRT, both at 3.0 T MRI and 1.5 T MRI, as follows:

(2) $$\begin{array}{c} ∆\text{SI}={\text{SI}}_{\text{pre}‐\text{CRT}}-{\text{SI}}_{\text{post}‐\text{CRT}}. \end{array}$$

### 2.5. Statistical Analysis

Statistical analysis was performed using MedCalc Statistical Software version 17.9.7 (MedCalc Software bvba, Ostend, Belgium).

All data are expressed as mean ± standard deviation (SD). Data distribution was assessed with the Kolmogorov-Smirnov test. In case of Gaussian distribution, data were tested with Student's *t-*test, while Wilcoxon's test was applied for non-Gaussian distributed data. A two-sided *P* value < 0.05 was considered statistically significant.

## 3. Results

### 3.1. Patient Population

**Figure 2:**
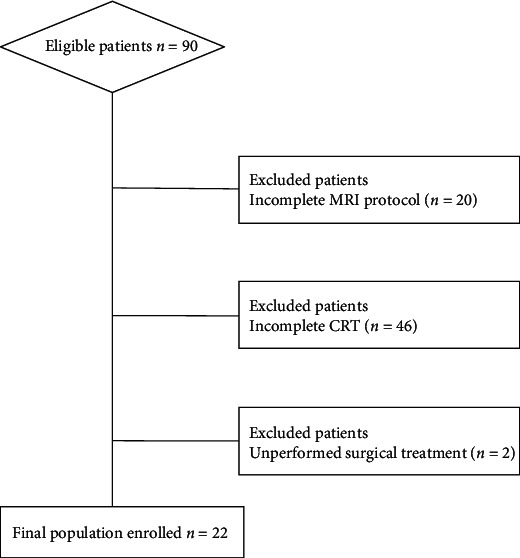
Flowchart of patients' selection.

Detailed patient characteristics are reported in Table 2. From an initial population of 90 patients, 68 were excluded due to incomplete MRI protocol at the time of the patients' selection (*n* = 20), noncompletion of the CRT at the time of the enrollment for the present study (*n* = 46), and unperformed surgical treatment (*n* = 2). Thus, the final population consisted of 22 patients (11 men; mean age: 65.59 ± 8.72; age range 48-81), as shown in the flowchart below (Figure 2).

On the basis of the MRI scans, 1 patient (4%) was diagnosed with local tumor staging of T2, 14 patients (63%) were diagnosed with T3, and 7 (31%) had a T4 rectal cancer, following the radiological staging UICC 2009 of I for 1 patient, IIA for 6 patients, IIIB for 9 patients, and III C for 6 patients.

All patients underwent total mesorectal excision. The pathological evaluation found complete response in 9 patients (41%), partial response in 5 patients (23%), and no response in 8 patients (36%).

### 3.2. Image Analysis

Full results are reported in Tables 3, 4, and 5. 3.0 T MRI provided significantly higher tumor SI at T2w sequences (all *P* ≤ 0.001), and DWI (all *P* < 0.001) compared to 1.5 T at each imaging control, while no statistically significant differences were reported for ADC values between 3.0 T and 1.5 T (all *P* ≥ 0.41). In addition, no statistically significant differences were found between 3.0 T and 1.5 T in terms of rSI for T2w (all *P* ≥ 0.11), DWI (all *P* ≥ 0.78), and ADC maps (all *P* ≥ 0.07) at each imaging control.

**Figure 3:**
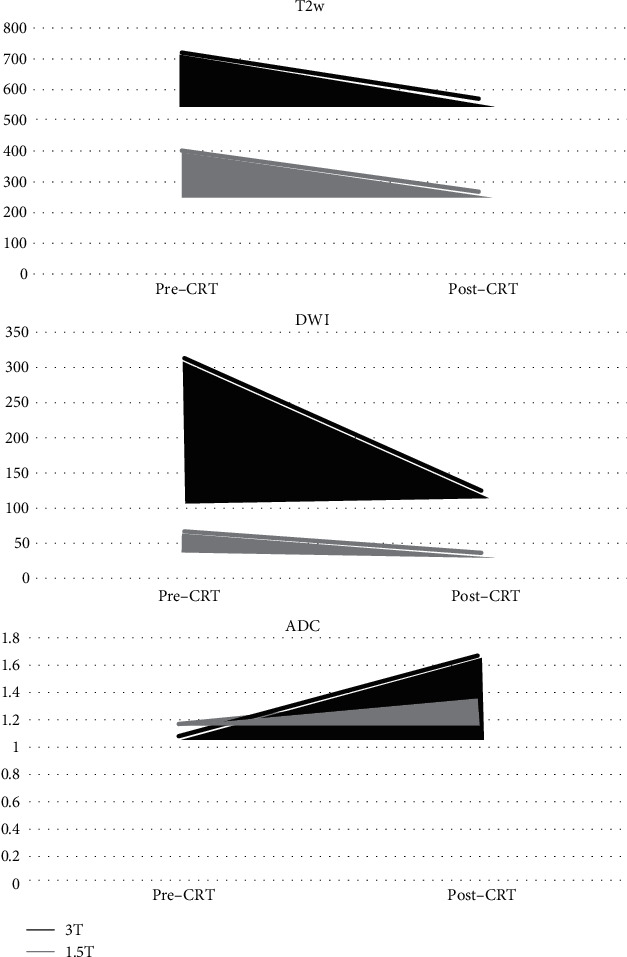
Graphics show the similar amplitude of *Δ*SI for T2w between 3.0 T and 1.5 T and the wider *Δ*SI for DWI and ADC on 3.0 T compared to 1.5 T.

The subanalysis performed comparing *∆*SI on 3.0 T MRI and 1.5 T MRI in complete responder patients (Figure 3) showed significant differences for DWI-*∆*SI (188.39 ± 166.90 vs. 30.45 ± 21.73; *P* = 0.02) and ADC-*∆*SI (−0.58 ± 0.27 vs. −0.21 ± 0.24; *P* = 0.02), while no significant differences were observed for T2w-*∆*SI (149.89 ± 211.92 vs. 133.64 ± 116.80; *P* = 0.87). The subanalysis of *∆*SI between 3.0 T and 1.5 T was also performed for NR showing significant differences for DWI-*∆*SI (188.39 ± 166.90 vs. 30.45 ± 21.73; *P* = 0.02); no significant differences were observed for ADC-*∆*SI and T2w-∆SI (−0.58 ± 0.36 vs. −0.44 ± 0.25 and 57.15 ± 224.02 vs. −57.26 ± 133.12, respectively; all *P* > 0.31).

## 4. Discussion

This study quantitatively compared SI on 3.0 T MRI and 1.5 T MRI in patients with rectal cancer before, during, and after CRT, aimed at assessing the impact of magnetic field strength in the diagnosis and follow-up. Our results showed that 3.0 T scanner provided significantly higher SI on T2w and DWI compared to 1.5 T scanner, while no significant differences in terms of ADC maps have been reported. Interestingly, the two scanners provided similar results in terms of relative signal intensity at every sequence. A possible explanation of this phenomenon could be the fact that higher magnetic fields are more susceptible to motion artifacts compared to lower magnetic fields. In fact, the absolute signal intensity can be influenced by many external and intrinsic factors, such as rectal peristalsis in the pelvic region. Thus, the signal normalization to a stable landmark, such as the obturator internus muscle, making rSI more standardized compared to absolute SI values [16, 17]. These results may lead to an important consideration: the added value of 3.0 T MRI scanner compared to 1.5 T MRI scanner in the evaluation of rectal cancer signal intensity may be questionable, also in accordance with the latest recommendations of the European Society of Gastrointestinal and Abdominal Radiology [18], which do not identify any of the two magnetic fields as the recommended imaging modality in the non-invasive assessment of rectal cancer. Interestingly, similar conclusions have been drawn by Ullrich et al. [17] for prostate MRI examination. The authors demonstrated similar objective image quality for T2w images at 3.0 T and 1.5 T scanners, ultimately leading to comparable diagnostic performances and Prostate Imaging Reporting and Data System (PI-RADS) score at both field strengths. Regarding a similar study on rectal cancer, Cai et al. [16] reported significantly higher diagnostic performance for normalized DWI at 3.0 T instead of 1.5 T; however, their results cannot be compared with the ones from our study that was focused on quantitative evaluation of SI and rSI instead of diagnostic performance.

The *∆*SI analysis performed in patients with pathologically proven complete response has demonstrated that 3.0 T MRI scanner has returned a significantly more pronounced *∆*SI for DWI and ADC maps in comparison to 1.5 T MRI scanner. Significant difference was observed also for *∆*SI-DWI in nonresponders between the two field strengths. It is well established that T2w SI is associated with intracellular water content [19–21] and that a high T2w SI reflects tumor viability [22]. The progressive signal drop characterizing the T2w sequences during CRT and after the completion of neoadjuvant treatment can be explained by the successful treatment itself, which is responsible for a reduction of viable tissue and a concurrent increase of fibrosis [23]. The concomitant increase of ADC values assessable at the end of CRT reflects the cellular density decrease and the interstitial space increase [24, 25] and gives further strength to this hypothesis.

**Figure 4:**
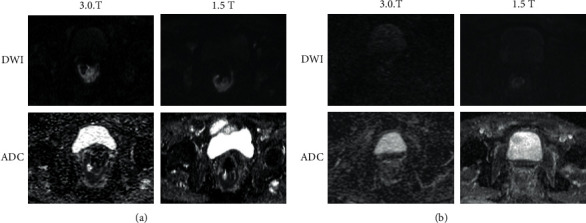
The picture shows DWI and ADC maps in CR at both field strength pre-CRT (a) and post-CRT (b). In post-CRT, on 3.0 T, the fibrotic tissue is better emphasized with no restriction of diffusion compared to 1.5 T where the difference with pre-CRT is less highlighted. In addition, on DWI, post-CRT at 1.5 T is more evident because of the shine through artifact of the rectal wall that could be misled as persistent viable tumor, while on 3.0 T is less evident despite the higher noisiness of the image.

Our results indicate that 3.0 T MRI scanner may outperform the 1.5 T MRI system in the visual assessment of rectal cancer in complete responder patients. Despite Maas et al. [15] demonstrating that 3 T and 1.5 T reached the same performance in rectal cancer staging, in our study, the higher *∆*SI of DWI and ADC of 3.0 T between the beginning and the completion of CRT in such patients may ultimately increase the visual assessment and readers' confidence in ruling out the presence of residual viable tissue at the end of CRT. On the other hand, at 1.5 T MRI examinations, a minimal residual restriction could be misdiagnosed as incomplete response to therapy (Figure 4).

This investigation should be evaluated taking into account some limitations. First, this preliminary investigation had a small sample size and is aimed at comparing quantitative MRI parameters in rectal cancer. The assessment of diagnostic performances was beyond our study design and further large prospective trials are advisable. Second, image analysis was performed on a single slice rather than on the whole tumor volume. Third, the comparative study was performed between two different vendors. Forth, due to the consensus reading of the selected slices, interreader agreement could not be performed.

In conclusion, despite significant differences of SI on T2w and DWI between 3.0 T and 1.5 T, the relative SI analysis showed similar results for both field strengths; therefore, per single examination, rectal cancer patients can be assessed both at 3.0 T MRI and 1.5 T MRI. However, DWI-*∆*SI and ADC-*∆*SI on 3.0 T reveals better results compared to 1.5 T in the response to treatment assessment and should be further investigated in a larger cohort of patients to verify this result and its possible clinical application.

## Figures and Tables

**Table 1:** MRI protocol for rectal cancer.

	Axial T2-weighted (T2w)		Axial diffusion-weighted imaging (DWI)	
Field strength	1.5 T	3.0 T	1.5 T	3.0 T
TR(ms)/TE(ms)	3380/90	2086-4172/11.4-122.3	6000/75	4400/81.4
Matrix	366 × 448	512 × 512	168 × 224	256 × 256
Number of signal average	2	2	2	2
*b* values (s/mm^2^)	—	—	0, 50, 400, 800, 1000	0, 200, 600, 800, 1000
Slice thickness	4	4	4	5

**Table 2:** Patient characteristics.

Total no. of patients	22
Gender, no.	
Female	11
Male	11
Mean age, yr ± SD (range)	65.59 ± 8.72 (48-81)
Radiological staging	
Stage I	1
Stage IIA	6
Stage IIIB	9
Stage IIIC	6
Histopathological features	
Adenocarcinoma	22
Mucinous adenocarcinoma	0
Pathological response to treatment (%)	
pCR	9 (41)
pPR	5 (23)
pNR	8 (36)

pCR: pathological complete response; pPR: pathological partial response; pNR: pathological nonresponse.

**Table 3:** Signal intensity values on 3.0 T and 1.5 T pre-, during, and post-CRT.

Signal intensity values									
	SI T2w			SI DWI			SI ADC		
	3.0 T	1.5 T	*P*	3.0 T	1.5 T	*P*	3.0 T	1.5 T	*P*
Pre-CRT	632.60 ± 183.02	341.88 ± 120.98	<0.0001^∗^	321.32 ± 176.99	70.77 ± 21.44	<0.0001^∗^	1.05 ± 0.22	1.07 ± 0.26	0.75
During CRT	511.85 ± 193.59	318.23 ± 107.77	0.0011^∗^	184.35 ± 95.47	51.47 ± 17.00	<0.0001^∗^	1.40 ± 0.28	1.33 ± 0.30	0.51
Post-CRT	525.08 ± 149.67	293.68 ± 99.17	<0.0001^∗^	136.33 ± 70.03	43.52 ± 14.84	<0.0001^∗^	1.55 ± 0.35	1.45 ± 0.32	0.41

^∗^Significant *P* values.

**Table 4:** Relative signal intensity values on 3.0 T and 1.5 T pre-, during, and post-CRT.

Relative signal intensity values									
	rSI T2w			rSI DWI			rSI ADC		
	3.0 T	1.5 T	*P*	3.0 T	1.5 T	*P*	3.0 T	1.5 T	*P*
Pre-CRT	2.99 ± 0.77	4.60 ± 4.84	0.14	4.80 ± 1.71	4.84 ± 1.40	0.95	4.90 ± 12.75	2.16 ± 0.98	0.07
During CRT	2.91 ± 1.24	3.42 ± 1.15	0.11	3.30 ± 0.95	3.37 ± 1.01	0.79	1.70 ± 0.99	2.02 ± 0.87	0.18
Post-CRT	2.30 ± 0.93	2.73 ± 1.03	0.14	2.91 ± 1.43	2.48 ± 0.76	0.78	1.85 ± 0.95	1.97 ± 0.73	0.16

^∗^Significant *P* values.

**Table:** (a) Complete responder patients

*Δ*SI pre-post-CRT							
	3.0 T			1.5 T			
	Pre-CRT	Post-CRT	*Δ*SI	Pre-CRT	Post-CRT	*Δ*SI	*P*
T2w	720.38 ± 232.91	570.49 ± 138.21	149.89 ± 211.92	401.61 ± 163.09	267.97 ± 120.55	133.64 ± 116.80	0.87
DWI	313.23 ± 181.74	124.84 ± 21.54	188.39 ± 166.90	66.73 ± 27.03	36.29 ± 8.21	30.45 ± 21.73	0.02^∗^
ADC	1.08 ± 0.24	1.67 ± 0.33	−0.58 ± 0.27	1.17 ± 0.28	1.38 ± 0.28	−0.21 ± 0.24	0.02^∗^

^∗^Significant *P* values.

**Table:** (b) Nonresponder patients

*Δ*SI pre-post CRT							
	3.0 T			1.5 T			
	Pre-CRT	Post-CRT	*Δ*SI	Pre-CRT	Post-CRT	*Δ*SI	*P*
T2w	559.42 ± 128.60	502.28 ± 176.81	57.15 ± 224.02	283.21 ± 66.36	340.47 ± 96.31	−57.26 ± 133.12	0.31
DWI	291.18 ± 140.89	121.48 ± 53.70	169.70 ± 121.87	69.15 ± 20.35	47.15 ± 17.99	22.00 ± 31.29	0.02^∗^
ADC	1.04 ± 0.19	1.62 ± 0.33	−0.58 ± 0.36	1.12 ± 0.22	1.56 ± 0.23	−0.44 ± 0.25	0.46

^∗^Significant *P* values.

## Data Availability

The data used to support the findings of this study are included within the article.

## Abbreviations

ADC: Apparent diffusion coefficient maps
CR: Complete responder patients
CNR: Contrast-to-noise ratio
DWI: Diffusion-weighted imaging
FOV: Field of view
2D FRFSE: High-resolution T2-weighted fast recovery fast-spin echo sequence
MRI: Magnetic resonance imaging
CRT: Neoadjuvant chemoradiotherapy
NR: Nonresponder patients
PR: Partial responder patients
RF: Radiofrequency
ROI: Region of interest
rSI: Relative signal intensity
SI: Signal intensity
SNR: Signal-to-noise ratio
SD: Standard deviation
T2w: T2-weighted
T: Tesla.
